# Experiences of shared decision making among patients with psychotic disorders in Norway: a qualitative study

**DOI:** 10.1186/s12888-022-03849-8

**Published:** 2022-03-17

**Authors:** Espen W. Haugom, Bjørn Stensrud, Gro Beston, Torleif Ruud, Anne S. Landheim

**Affiliations:** 1 Norwegian National Advisory Unit on Concurrent Substance Abuse and Mental Health Disorders, Innlandet Hospital Trust, P.B 104, 2381 Brumunddal, Norway; 2 Norwegian Centre for Addiction Research (SERAF), Institute of Clinical Medicine, University of Oslo, Oslo, Norway; 3 Division of Mental Health Services, Akershus University Hospital, Lørenskog, Norway; 4 Institute of Clinical Medicine, University of Oslo, Oslo, Norway; 5 Department of Public Health, Inland Norway University of Applied Sciences, Elverum, Norway

**Keywords:** Shared decision making, psychotic disorders, mental health services, qualitative research

## Abstract

**Background:**

Shared decision making (SDM) is a process where the patient and the health professional collaborate to make decisions based on both the patient’s preferences and the best available evidence. Patients with psychotic disorders are less involved in making decisions than they would like. More knowledge of these patients’ experiences of SDM may improve implementation. The study aim was to describe and explore experiences of SDM among patients with psychotic disorders in mental health care.

**Methods:**

Individual interviews were conducted with ten persons with a psychotic disorder. They were service users of two community mental health centres. The transcribed material was analysed using qualitative content analysis.

**Results:**

Four-fifths of the participants in this study found that they received insufficient information about their health situation and treatment options. All participants experienced that only one kind of treatment was often presented, which was usually medication. Although the study found that different degrees of involvement were practised, two thirds of the participants had little impact on choices to be made. This was despite the fact that they wanted to participate and felt capable of participating, even during periods of more severe illness. The participants described how important it was that SDM in psychosis was based on a trusting relationship, but stated that it took time to establish such a relationship.

**Conclusions:**

This study with ten participants indicates that patients with psychotic disorders experienced that they were not allowed to participate as much as they wanted to and believed they were capable of. Some patients were involved, but to a lesser degree than in SDM. More and better tailored information communicated within a trusting relationship is needed to provide psychotic patients with a better basis for active involvement in decisions about their health care.

## Background

Service user involvement has been emphasized as a crucial aspect of contemporary mental health care in recent decades [1]. The World Health Organization supports this by promoting patient involvement and empowerment as a means to improve health care [2]. In the pathway for mental health and substance abuse in Norway, a key goal is greater service user participation and satisfaction [3]. Moreover, hospital trusts in Norway are required to ensure that mental health care patients, as far as reasonably possible, can choose between different treatment options, including treatment without medication [4]. Despite this, a recent evaluation of the mental health care pathway shows that few patients receive information about different treatment alternatives and that even fewer find they can choose their treatment [5].

Shared decision making (SDM) has been introduced as an approach where patients and health professionals collaborate to make decisions based on patients’ preferences and the best available evidence [6]. This approach is in the middle of a continuum from patient-led to clinician-led decision making [6, 7], and may improve patient satisfaction [8, 9] and provide less decisional conflict [9]. SDM is closely related to both user participation and empowerment, as it aims to strengthen patient autonomy [6] and facilitate people’s right to make health care decisions [10]. The practice of SDM also reflects essential elements in person-centred care, e.g. that patients’ views should guide all clinical decision making [11, 12]. These are all key elements of the recovery process, and thus SDM reflects the values of recovery-oriented mental health care [13] and is a central part of the recovery process [14, 15]. A recent study also indicates that SDM may improve personal recovery [16]. Further, although it is not the same, SDM has also been associated with the concept of co-production, as they both recognize that improvement in health care quality requires involvement of the care recipients [17, 18].

Several conceptual frameworks, models and definitions have been suggested [19–21] since SDM was introduced in the 1980s [22]. Makoul and Clayman [20] describe how SDM includes presenting information about the patients’ health issues and treatment options, and discussing pros and cons and the patient’s ability/self-efficacy. Further, the health professional presents a recommendation, checks and clarifies the patient’s understanding, before the patient and the professional make the decision or explicitly defer it until a later meeting. If a decision has been reached, follow-up will be arranged to track the outcome of the decision. All of these actions are guided by the patients’ values and preferences.

Conceptual frameworks for SDM in the mental health care setting have also been suggested [23–25]. Morant et al. [24] propose a conceptual framework that moves beyond the micro-social process. They emphasize that a broader model is necessary in order to view SDM as a variety of related processes not only within, but also beyond a single psychiatric consultation. Gurtner et al. [23] support this as they view SDM as a process that is usually not limited to a single consultation. This is important because mental illness often involves long-term treatment and thus requires that the patient and health professional interact in relationships over time [24]. This can be seen in the context of another study that finds that additional elements such as building a trusting relationship are important and necessary to practise SDM in mental health care [25].

The SDM model is recommended at the policy level and is advocated as the ethically right thing to do [7]. Moreover, decision aids have been developed to facilitate SDM [26, 27]. However, implementation in mental health care remains limited [7]. Health professionals mention reduced decisional capacity as a barrier to using SDM with patients with psychotic disorders [28, 29]. Barriers among patients with schizophrenia may be experiences of powerlessness due to coercive treatment that even many years later prevent them from expressing their ideas and preferences [30]. Further, the fear of coercive measures prevents mental health service users from reporting symptoms and is a barrier to involvement in medication decisions [24].

Interview studies on SDM experiences among patients in mental health care with different diagnoses have found that they considered SDM as relevant to all aspects of inpatient and outpatient care [31]. It has also been found that patients want all health professionals involved in their care to cooperate and be complementary to their own participation in SDM [32]. Despite this, another interview study showed that mental health care patients struggle for recognition as competent and equal partners in decision making, which may reflect an imbalance in the decision-making process [33]. This is also evident in an interview study of user involvement and medication treatment among patients with psychotic disorders. They found that patients wanted more information and dialogue about their diagnosis, greater participation in both pharmacological and other treatment options, and medication to be part of a holistic treatment regimen [34]. Further, patients with schizophrenia reported in an interview study that they were insufficiently involved in treatment-related decisions despite their desire for SDM. These patients also felt that even in periods of decision-making capacity they were not actively involved in decisions. They considered that improved education and training, holistic care, and being viewed as people with expertise can facilitate SDM [8].

The challenges in implementing SDM in mental health care call for further research [7, 35]. Although studies have investigated involvement of patients with psychotic disorders and SDM for patients in mental health care, fewer interview studies have investigated SDM experiences exclusively for patients with psychotic disorders. The fact that patients with psychotic disorders value being involved in decisions regarding their treatment [8, 36], but are less frequently involved than other patient groups in mental health care [37, 38], shows that more studies on this patient group are necessary.

This study attempts to add new knowledge to the field by exploring SDM from the perspective of people with psychotic disorders and many years’ experience in mental health care who mainly receive outpatient treatment and live in the community. Examining these patients’ experiences of SDM may help us to understand better how to empower them to collaborate in making health care decisions. This may be a valuable contribution towards providing more recovery-oriented services. The study aim was to describe and explore experiences of SDM among patients with psychotic disorders in mental health care.

## Methods

### Design

The study was a descriptive and exploratory qualitative study with an inductive approach [39] using Graneheim and Lundman’s qualitative content analysis [40]. This method focuses on the subject and the context in addition to differences between and similarities within codes and categories. The study was conducted in collaboration with a person with prior service user experience. The current study is an independent part of a larger project investigating the implementation of evidence-based practices for patients in psychosis treatment [41].

### Recruitment and setting

The sampling strategy was purposive and aimed to recruit participants who were expected to provide the most information about experiences of SDM of persons with a psychotic disorder.

The participants were recruited from two community mental health centres (CMHCs) involved in the larger project [41]. Both CMHCs aimed to implement an evidence-based model of antipsychotic medication. Implementing the antipsychotic medication model contained SDM and nine other components [42]. The implementation phase was from 1 September 2016 to 1 February 2018.

Health professionals from the CMHCs gave patients information about the current study and invited them to participate based on the following inclusion criteria: the participant was receiving or had received treatment at the CMHC, had participated in the larger implementation project, had consented to be invited to participate in a follow-up study, was > 16 years old, and had a psychotic disorder (ICD-10, F20-29).

At the first CMHC, the leader coordinated the recruitment process and made appointments for interviews in consultation with the first author. At the second CMHC, the health professionals provided the phone number of those participants who agreed to be interviewed to the first author, who then made appointments for interviews.

The study was conducted in two CMHCs in the east and north of Norway. The services these CMHCs provided included acute psychiatric units, units for treatment of psychotic disorders and different outpatient units. The catchment areas of the CMHCs included cities, smaller towns, and rural regions. CMHCs have multidisciplinary teams which may consist of nurses, social workers, occupational therapists, psychiatric nurses, social educators, psychologists and psychiatrists. They all have individual treatment responsibilities. However, a psychologist or psychiatrist has the overall responsibility. When a patient receives medical treatment, a psychiatrist will always be involved.

### Participants

Ten participants meeting the inclusion criteria consented to participate, four from the first CMHC and six from the second one. Six women and four men were included in the sample. Their ages ranged from 32 to 72 years, with a median age of 53. All participants had a psychotic disorder.

Eight participants received voluntary outpatient care at the time of the interviews, one received voluntary inpatient care and one was not receiving any care at all. Six participants had previous experienced compulsory mental health care. Nine had experienced using antipsychotic medication. Seven participants lived in their own home, while three lived in supported housing.

The participants’ first contact with mental health services had taken place from five to 49 years ago (median 25 years). Half of the participants in treatment were mainly receiving care from a psychiatrist. The remainder mainly received care from other health professionals, but had a psychologist or psychiatrist with overall responsibility for their treatment. Considering that nine participants had experience of taking antipsychotic medication, it is reasonable to assume that those who received care from other health professionals also had some contact with a psychiatrist.

### Data collection

The data were collected from ten individual interviews, one with each participant. The research group, which included the third author who had prior service user experience, designed a thematic interview guide (Table 1). The questions were open with little structure and aimed to let the participants tell their story freely.

**Table 1:** Thematic guide for interviews with patients

**Main topic** - Patients’ experiences of shared decision making	
**Introductory question:** - Can you tell me about a recent meeting with your health professional?	
**Sub-topics**	
- Experiences of how decision making is practised	
- Understanding of the shared decision making concept	
- Advantages versus disadvantages of practising shared decision making	
- Enablers and barriers to shared decision making	

The first author conducted the interviews from December 2019 to March 2021. Seven participants were interviewed at the CMHC, two were interviewed in their homes, and one was interviewed in a meeting room at the first author’s workplace. Only the interviewer and the participant were present during the interviews, which lasted from 43 to 72 minutes (median 54 minutes).

### Data analysis

After each interview, the first author wrote a short text to summarize impressions and reflections. All interviews were audio recorded and transcribed verbatim by the first author. The transcribed interviews were read several times to become familiar with the material. The authors identified meaning units in the text, i.e. words, sentences or paragraphs related to each other through their content and context [40]. The meaning units were labelled with a code, and condensation involved writing the meaning units in a shorter form while preserving the core meaning.

The codes were discussed and compared for differences and similarities, and then sorted into categories describing the content on a manifest level [39]. A recurrent theme running through the categories was identified through interpretation of the latent content. The theme and categories were compared with the interview transcript to ensure that they covered the experiences of SDM as described by the participants [40]. The categories were presented using representative quotations from the participants to illustrate the findings. The development of categories and the theme were regularly discussed with the second, third (the researcher with prior service user experience) and last authors to validate the understanding. Proportions are used presenting the results. Not all participants had the opportunity to respond to all questions, as questions being asked depended on the stories told by the participants and how the interview progressed. This explains why proportions such as one third and two thirds are used. The categorization of findings was supported using NVivo 12 Pro.

### Ethical considerations

A psychotic disorder may affect a person’s ability to receive and process information, and thus challenge the capacity to give informed consent to participate in research. However, this should not be sufficient reason to deprive a group of people of the opportunity to promote their own views. Health professionals assessed the participants’ decision-making capacity, and excluded only those obviously unable to understand what consent to participate in the study entailed. The assessment of decision-making capacity involved assessing the patients’ ability to understand the information relevant to the decision, to recognize how this information was relevant in their unique situation, to reason using relevant information when weighing up the options, and to communicate their choice [43]. The participants were informed orally and in writing about the study before signing a consent form. All participants gave fully informed consent voluntarily. They all had the opportunity to contact a health professional after the interview if they needed counselling or other assistance. The study was approved by the Regional Committee for Medical and Health Research Ethics (REC South-East, Reg. No 2015/2169). Approval was also obtained from the data protection officer of each participating health trust.

## Results

The main finding of the study, “*able to collaborate, but not always involved on their own terms*”, reflects that the participants felt capable of participating in decision making but also found that health professionals did not always involve them as much as they wanted. The main finding is presented below through the following four categories: *participation is desirable and achievable, shared decision making requires a trusting relationship, insufficient information,* and *varying degrees of involvement.*

### Participation is desirable and achievable

Four fifths of the participants felt that it was important to participate in SDM. They stated that it was important for their voice to be heard in the decision-making process by being allowed to take part in a discussion where the goal was to reach a shared solution. The participants mentioned that in their bad periods they were still able to participate in SDM if invited to do so.

Interviewer: *“What do you think when you’re in a really bad period? What do you think about participating then?”*

Participant 4: *“Yes. I reckon I could do it if I was asked a bit more. If it was arranged like that (…) then I think I’d be able to, well, be more involved.”*

The participants considered it important to be heard also when they were in a more severe phase of their illness. One said that cooperation was vital if he became worse, explaining that he could think quite clearly and was able to cooperate even when he was psychotic. Another participant reported that she had the strength to think and make decisions when she was psychotic.

Participant 5: *“Your thoughts are clear enough so you can manage to understand and make a decision.”*

Although participants stated that participation was desirable and achievable even when their symptoms deteriorated, there were also one third of the participants who felt that the professionals should take the decision if they became so ill that they did not understand the consequences of their actions.

### Shared decision making requires a trusting relationship

Participants found it important to be able to trust the therapist when cooperating. This is illustrated by the dialogue below between the interviewer and a participant who experienced good collaboration with his therapist.

Interviewer: *“Could you say a little about what you think is the reason that you have such good cooperation?”*

Participant 7: *“Well, I reckon it’s because I’ve always been able to trust her. It’s never happened that she hasn’t kept what she promised. That’s why I can really rely on her, and she’s helped me through lots of difficult periods.”*

One third of the participants mentioned that they felt secure when they could trust their therapist. Two thirds of the participants stated that it was important to be listened to and taken seriously, and that this could help to build a relationship based on trust. One said that when he was taken seriously, it meant that he was finally understood. Another described how it was easier to be involved and make choices when the professionals listened to him because then no decisions were made over his head. The participants reported that a sense of being seen and heard made them dare to open up about how they were feeling.

One third of the participants stated that a relationship based on trust had to be built over time and that this could take years. An important topic was the question of finding professionals where there was good chemistry. This meant someone you got along with, a good match or “the right one”, as one participant stated.

Participant 6: *“It’s about getting... Getting a person where there’s good chemistry. Someone you can trust so you can tell them how you’re feeling. You have to feel that you, like, you have a kind of, you know, trust, that you... I mean that you can trust someone and you feel you can talk to them. Because you don’t get on in the same way with everybody. When you want to... So it’s good to get a therapist or someone you get along with.”*

But the participants also said that it was not easy to find such a person. A couple of participants reported having found “the right one”, but said that they had been to a number of therapists before they met this person.

### Insufficient information

Four-fifths of the participants reported receiving little information about their health situation and various treatment options.

Participant 9: “*I wouldn’t say they’re absolutely brilliant at giving me information about my illness. I had to dig deep to get hold of it, you know. So there’s maybe too much secrecy about it.”*

An important topic was that professionals presented a single treatment option, which was generally medication.

Participant 2: *“Mostly it’s been like one type* [of medicine] *has been suggested, then we talked a bit and then I took it. I don’t know that much about these medicines.”*

The participants were told by their therapists to let them know if a medicine did not make them feel better or if they had side effects, to enable the medication treatment to be adjusted. A crucial issue was that the participants reported having to try different medicines before they found one that helped them.

A participant were unsure of how many other treatment options there were in addition to medical treatment.

Participant 3: *“I really don’t know how many different kinds of treatment there are for something.”*

One said he did not know what kind of medication he was taking. Another reported having received information about how effective the medicine should be, but she was never given any information about side effects.

Participant 5: *“They ought to be better at telling you about side effects.”*

Interviewer: *“So they haven’t told you so much about that?”*

Participant 5: *“No… Just how good it is for your head and your mind and so on, but not the side effects.”*

Four fifths of the participants found that the information they received was not tailored to their situation. Although they called for more and better tailored information, one fifth of the participants also said that they had discussed their health situation with the therapist and received helpful information about medications.

Participants who initially stated that they were only offered medical treatment later revealed in the interviews that they had also received other forms of treatment, such as counselling and physical activity. However, these had not been presented as alternatives to medication treatment.

### Varying degrees of involvement

Two third of the participants described how treatment decisions were made by professionals and said that they had to accept the alternative presented.

Participant 6: *“I’ve been bulldozed into it. People have made the decisions for me. I haven’t been asked that much about what I think and so on. So it’s a bit difficult. But I’ve never had that cooperation or the* [shared decision making]*. So I feel that’s something I actually miss in the therapy situation.”*

One participant had the experience of being pathologized and not taken seriously, and believed she had given up her right to cooperate in treatment options. Another participant expressed frustration when his voice was not heard in the decision-making process.

Participant 2: *“I feel I’ve had very little say in the decisions. It’s like, deep inside me there’s a whole lot of frustration. I read something about shared decision making, then I thought, oh my God, shared decisions, I haven’t seen much of that.”*

Participants reported that during voluntary treatment they were not listened to when they wanted to change their medication. One participant was tired of being met with a “diagnosis focus” and an expectation that his condition would deteriorate if he stopped taking medicine. He was never given the chance to try coming off medicine. An important topic was that participants missed being invited to take part in decisions when they felt better, and were annoyed that decisions made when they were in a bad period were still valid for a long time afterwards.

However, the participants also had different experiences of SDM. One was allowed to change the method of administration from injections to tablets when she asked for this, and another was allowed to reduce her dose when she thought it was too large.

Participant 4: *“They put me on 40 mg Quetiapine. But I thought that the dose was too big. And I was allowed to reduce it.”*

One third of the participants reported discussing treatment and forms of treatment, where the therapist took their preferences into account and decisions were made in cooperation with the therapist. These participants stated that cooperation on decisions was a matter of finding the golden mean.

Participant 10: *“I’m pleased we make a compromise... I don’t get my way, and she doesn’t get hers. We just have to find a way that works.”*

One participant found that they needed to discuss until they reached an agreement where both parties had to give and take a little. Participants stated that it could take time to reach agreement; in that case they would agree to continue the discussion at a later date.

However, a couple of the participants who found that decisions were made with the therapist also said that they were too soft and tended to give in. They were afraid to make a different choice from the suggestion of the professional; the therapist knew best and had the last word in practice.

## Discussion

The present study showed that patients with psychotic disorders experienced that they were not always involved in line with their preferences and perceived capabilities. They often felt that they received little information about their health situation and various treatment options. A single treatment option was generally presented, which was mainly medication. This runs contrary to health policy guidelines on more person-centred and recovery-oriented health services, where SDM should be the norm. The results show that SDM is possible, but that it requires a treatment relationship based on trust.

An essential element of SDM is to provide patients with information about their health situation and various treatment options [20]. This study found that four fifths of the patients felt that they received inadequate information on this. This concurs with previous research showing that patients with psychotic disorders wanted more information and discussion about the diagnosis [34]. Similarly, the evaluation of the pathway showed that few mental health patients received information on treatment alternatives [5]. This finding is interesting in light of another study of the same CMHCs as those included in this study. That study found that health professionals felt that informing patients about their health situation was an important aspect of SDM [29]. This may mean that patients and professionals at the same CMHCs have different experiences of the degree to which information is provided. Our study showed that the patients found that they had generally not received sufficient information to form a basis for potential SDM. Other studies have found that both patients and health care professionals considered that information about the illness and treatment was an important facilitator of SDM [44, 45], and that information may be the key to SDM [28]. The present study supports this and emphasizes the importance of personalized information in achieving SDM.

Previous research has shown that antipsychotic medication dominates in the treatment of psychotic disorders [46, 47]. In our study, the patients found that the professionals mainly presented information about a medicine when deciding on treatment. This is in line with a study that showed that health care professionals at the same CMHCs from which we recruited patients felt that SDM was about choosing antipsychotic medicine [29]. A previous study found that health care professionals sometimes decided not to inform patients about alternatives to the treatment they considered most relevant [33]. Similarly, the present study found that patients were mainly presented with a single recommended treatment. Patients with psychotic disorders want medication to be part of a holistic treatment approach [34]. A holistic approach recognizes the person’s whole life as important and includes non-medical aspects [48]. This is in line with a review that revealed a need to develop more SDM interventions for options such as deciding about rehabilitation services and psychosocial programmes [25]. We found that patients received care in other areas, but this was presented as a supplement, not an alternative, to medication treatment. This is interesting since patients in Norway are entitled to choose medication-free treatment [4]. The study showed that a holistic approach was not widely practised. This may suggest that the patients we interviewed mainly received treatment where the goal was clinical recovery, i.e. with a greater focus on treatment and symptom reduction than the patient’s subjective experience of personal recovery [49].

One possible explanation for why the participants focused on medication may be that they related the experiences they shared in the interviews to medicine consultations with psychiatrists. It is also important to interpret the focus on medication in relation to current guidelines for psychosis treatment. These recommend treatment with antipsychotic medication and state that psychological interventions are most effective when combined with medication [50]. The Norwegian health authorities and service user organizations have focused strongly on treatment options without medication in recent years [4, 51]. However, we did not find evidence of these efforts in our study.

The study showed that although different degrees of involvement were practised, two third of the patients found that they generally had little real decision-making authority. This is in line with previous studies that found that SDM was little practised in mental health care [7], and even less with psychotic patients than other patient groups [37, 38]. This is noteworthy since four fifths of the participants in this study wanted to be involved in decision making, and believed that they were capable of this even in periods of increased symptoms. This is interesting in light of findings from a recent study that found that patients with schizophrenia experienced that even in periods of decision-making capacity, they were not actively involved in decisions [8]. Further, our finding can be seen in connection with previous research showing that a large proportion of people with psychotic disorders have adequate decision-making capacity [52–54]. Most of the participants in our study were receiving outpatient treatment, and thus probably had higher levels of functioning and greater capacity to make decisions than inpatients with psychotic disorders. However, a further important factor is that six of the interviewees had experience of involuntary admission. This may indicate that they had sometimes needed the help of health care personnel in decisions on their health. A couple of participants also expressed this in the interviews.

The fact that more than half of the participants had been involuntarily admitted to hospital may have influenced the kind of experiences they shared in the interviews, and thus may explain why they reported little participation in SDM. Another explanation may be that the patients had been in contact with health care services over many years. This explanation is supported by previous research where patients who had been in contact with health care for a long time reported a lower degree of SDM than those with a shorter period of contact [38]. Many years of experience with a potentially clinician-led health service can be related to some participants’ descriptions of themselves as passive recipients of health care and may have contributed to their feeling of having little say in decisions.

Patients in treatment can take on a passive role for various reasons. A systematic review explored barriers to involving people with severe mental illness in antipsychotic prescribing [45]; some reasons for patient passivity were that they believed the professionals knew best, they were unaware of their right to participate, or they had previously been denied participation. These factors are in line with our study, and underline the importance of patients receiving information about why they should participate. SDM is appropriate in preference-sensitive decisions with more than one sensible treatment option and a choice that involves balancing advantages and disadvantages, where the patient’s values ​​are included in making the “right” decision [55, 56]. If patients are informed about this, it may give them a different view of their role. They may then realize that collaboration on treatment in line with their preferences is important to enable them to cooperate with health care personnel in creating an improved service where health care is adapted to patient values and wishes. At the same time, it is important to remember that it is up to patients to decide whether they want to participate, and that a decision not to participate must also be respected. They must be given enough information to understand their role in the decision-making process; this information should enable them to decide for themselves on participation. However, this study shows that this was only done to a limited extent.

The study findings reveal more of a clinician-led model than SDM. In a clinician-led model, collaboration is abandoned in favour of a relationship where the professional makes the decision for the patient [7]. Here, the decision-making process does not include two equal experts with equally important forms of competence as in the SDM model [55]. On the contrary, the competence of the professional carries more weight than that of the patient. This was clearly seen when participants said that the professionals knew best. It was also expressed by some participants who did not dare to choose a different treatment than that suggested by the professional. In such situations, health care professionals are in a position of power, while patients are left with no power and little freedom. They are thus not included in a discussion of possible treatment options with the aim of reaching a shared decision. Professional power in mental health care may also be seen in relation to the possibility of using coercion [57], and the example above can be seen in light of the term “coercive shadow”. This refers to patients accepting the treatment offered to avoid the humiliation of involuntary admission [58].

When patients feel unsure of themselves and lack the confidence to participate actively by communicating their preferences, an emphasis on SDM can help to restore patient self-determination [56]. If SDM is used in the context of person-centred care, it can become part of holistic treatment and care where help is offered in several areas. Person-centred care involves adapting the approach to the specific needs and values ​​of the individual patient. This is important if SDM is to be meaningful and useful for both patients and professionals [59]. Further, this can help to change the health service from a setting where the patient is a passive recipient of care to one where professionals and patients create the service together. The goal of treatment in a person-centred approach will be in line with the principles of personal recovery [60], which imply that the person should live a meaningful life [48]. SDM may enhance personal recovery [16], and a person-centred SDM approach can promote holistic aspects of recovery that were lacking in our data, such as decent housing, education, and employment in addition to the treatment patients already receive.

This study found that some patients reported receiving useful information and having a good dialogue and collaboration with their therapist, where their preferences were taken into account. Some stated that if the prescribed medicine did not have the desired effect or caused side effects, they were allowed to try an alternative. Clearly, this is important. However, a practice in line with the SDM model will mean that the benefits and drawbacks of different treatment options are discussed before a decision is taken [20]. The study showed that the treatment received by these psychotic patients did not reflect the SDM model as defined in the literature. Despite this, we found that some patients’ voices were heard in individual cases. They may have been involved to a certain extent, meaning that some specific wishes were respected. However, their values and preferences were not included in the entire decision-making process in line with the aim of SDM. This may suggest that patients are allowed to participate to a degree, but that health care services still have a long way to go to achieve the type of participation and collaboration described in SDM. At the same time, perhaps patients also have a long way to go to become an active party in decision making. This was seen in a previous study which showed that patients themselves can facilitate the use of SDM [30]. In order to take on an active role, however, they need to be provided with useful information about the importance of participating, their health situation and relevant treatment options, and to be empowered to participate in a collaborative effort to reach a common understanding where the patient’s values and preferences are addressed.

It is worth noting that the participants who described being involved generally received care and treatment from the same therapists. A previous Norwegian study found that whether patients felt they could influence treatment was dependent on their particular therapist [5]. This underlines the importance of the findings of this study that the possibility to achieve SDM lies in the therapeutic alliance, and that good chemistry with the therapist is important to develop the confidence and trust needed to implement SDM. This is supported by a study that found that building trusting relationships was considered necessary to practise SDM in mental health [25], and is in line with another study where service users with serious mental illness found that SDM required a long-term therapeutic partnership with honesty from both sides and an ongoing endeavour to build mutual trust [61]. These findings are also interesting in light of a previous study that found that a good therapeutic alliance is important in achieving recovery [62]. However, our study also showed that it takes time to build a relationship, and that it was challenging for the patients to find professionals where the chemistry was so good that they could establish a trusting relationship.

### Strengths and limitations

A strength of this study is that individual interviews with people with a psychotic disorder yielded rich and nuanced descriptions from a service user perspective. A further strength is that a person with prior user experience participated in the planning of the study, read all the transcripts, took part in the analysis process and gave critical input to the final manuscript. A final strength is that we had a relatively homogeneous sample in that the participants interviewed had many years’ experience of mental health care, mainly received outpatient treatment and lived in the community.

There are several study limitations. First, we did not return the transcripts to the participants for comments and/or corrections. However, summaries were used during the interviews to allow the participants to correct the researcher’s understanding and interpretation [63]. Second, the participants did not provide feedback on the findings. However, four researchers including a person with lived experience were involved in the analysis to ensure a reflexive process where we challenged each other’s preunderstandings in an effort to remain open and let the data reveal their uniqueness. Third, the study was conducted in a limited geographical area and thus may only represent local practices. Fourth, half of the participants mainly received care and treatment from their psychiatrist, and medication may have played a key role in this. In addition, other participants may have thought of their previous meeting with their psychiatrist when they were asked to talk about a recent meeting with health professionals. Further, we only interviewed participants who were treated at CMHCs that implemented evidence-based antipsychotic medications. These may be possible explanations for why medication played such a key part in the participants’ descriptions.

Data saturation was considered to occur after ten interviews as previous themes were confirmed without providing new perspectives. However, considering the small sample size, we cannot rule out that additional participants could have contributed more information about the phenomenon that was investigated. The aim of this study was not to generalize, but to describe essential experiences of the participants with SDM. The results may be transferable and relevant to other settings in mental health care involving patients with a psychotic disorder and their service providers.

## Conclusions

The fact that patients with psychotic disorders experienced that they received inadequate information about their health situation and various treatment options, while their views were generally not heard in decision-making situations, shows that shared decision making was not practised according to the model. If the patients were involved, it was at a lower level than in the shared decision making model, and not always to the extent that patients wanted and thought they were capable of. The study shows that the implementation of shared decision making requires a therapeutic relationship based on trust, but that this takes time to establish, and patients have great difficulty in finding health care professionals where the chemistry is so good that such a relationship is possible.

There is a need to focus more strongly on how health care services can provide more and better tailored information. Service providers should also aim to build trusting relationships. This can give patients a better foundation for active participation with professionals in decisions on their health care.

## Data Availability

The qualitative data material or parts of the data may be considered available from the first author Espen W. Haugom upon reasonable request. Contact details: Espen.Woldsengen.Haugom@Sykehuset-Innlandet.no

## Abbreviations

SDM: Shared decision-making
CMHC: Community mental health centres
